# Safety Culture and Multidisciplinary Teamwork in Paediatric Care Transitions: A Systematic Review

**DOI:** 10.3390/children13101387

**Published:** 2026-10-12

**Authors:** Agon Hoti, Leona Cilar Budler

**Affiliations:** Faculty of Health Sciences, University of Maribor, 2000 Maribor, Slovenia

**Keywords:** medication reconciliation, paediatric care transitions, medication safety, multidisciplinary teamwork, patient safety, interprofessional collaboration

## Abstract

**Highlights:**

**What are the main findings?**

• Organisational and team-based factors may influence medication safety and accuracy during paediatric care transitions.
• Pharmacist involvement in medication reconciliation was associated with improved medication accuracy across paediatric transitions of care.

**What are the implications of the main findings?**

• Integrating medication reconciliation with structured interprofessional collaboration may strengthen medication safety during paediatric care transitions.
• Standardised medication-reconciliation and communication processes may help reduce discrepancies at admission, transfer, and discharge.

**Abstract:**

**Objective:** This systematic review synthesises current evidence on the role of organisational safety culture and team-based approaches in medication safety and accuracy during paediatric care transitions. **Study Setting and Design:** A systematic review was conducted in accordance with PRISMA 2020 guidelines, focusing on studies that examined organizational or team-based approaches to medication safety and medication reconciliation at paediatric transition points, including admission, transfer, handover, and discharge. **Data Sources and Analytic Sample:** PubMed was systematically searched for studies published up to August 2026, identifying 108 records. After screening and applying predefined inclusion and exclusion criteria, 13 studies were included in the final synthesis. **Principal Findings:** Across heterogeneous study designs and paediatric transition settings, medication discrepancies were frequently identified at admission, transfer, and discharge. Pharmacist involvement, structured medication reconciliation, and standardised communication processes were recurrent components of interventions associated with improved medication-information accuracy. Because the evidence was predominantly observational and quality-improvement-based, findings were interpreted as associations rather than evidence of causality. **Conclusions:** The available evidence suggests that team-based approaches, particularly pharmacist involvement, structured medication reconciliation, and standardised communication processes, are associated with improved medication safety and accuracy during paediatric care transitions. Organisational support and clearly defined interprofessional roles may facilitate the implementation of these approaches. Further high-quality studies are needed to clarify their effectiveness across different paediatric transition settings.

## 1. Introduction

Transitions of care represent one of the most vulnerable points in the continuum of healthcare for paediatric patients. Movement from the hospital to home or to another healthcare setting is frequently accompanied by fragmented communication, incomplete documentation, inconsistent caregiver involvement, and time pressure. These challenges increase the likelihood of discrepancies between the medications a child is actually taking and those recorded in clinical documentation. Because paediatric pharmacotherapy relies on age- and weight-adjusted dosing, involves frequent regimen changes, and depends on coordination among multiple caregivers, the potential for medication errors during transitions of care is particularly pronounced. For these reasons, ensuring accurate medication information during transitions is especially critical in paediatric practice.

Despite the availability of transition frameworks, important gaps remain between recommended practices and their implementation in paediatric healthcare [1]. System-level barriers, including fragmented communication and limited continuity of care, may further compromise safe transitions from hospital to home and across healthcare settings, particularly for children with complex care needs [2]. Similar challenges related to continuity and coordination have also been reported during the transition from paediatric to adult healthcare services [3].

Structured medication reconciliation is an important patient-safety strategy during transitions of care, enabling healthcare professionals to review and reconcile medication information at admission, transfer, and discharge. In a paediatric quality-improvement study, Gunkelman et al. [4] found that interventions involving simplified EHR workflows, resident education, and enhanced accountability increased the proportion of patients with at least one unnecessary medication removed from the home medication list from 35% to 48%.

However, effective medication reconciliation extends beyond the technical process of comparing medication lists and may also depend on organisational and interprofessional factors. Stolldorf et al. [5] identified limited interprofessional communication, organisational capacity, and the definition of professional roles and responsibilities as important contextual factors influencing medication reconciliation implementation.

Paediatric operating room-to-intensive care unit handoffs further illustrate the importance of team communication during care transitions. Malenka et al. [6] evaluated a multidisciplinary standardised handoff protocol, including a bedside checklist, for transfers from the operating room to the paediatric intensive care unit. Following implementation, the completeness of information transfer increased from 56% to 81%, without a significant increase in handoff duration. These findings suggest that standardised, multidisciplinary handoff processes may improve the completeness and consistency of information transfer during paediatric care transitions. Recommendations arising from the study, including full-team participation in handoffs, early inter-unit communication, and technological support for structured information capture, directly reinforce the premise that safe paediatric transitions depend on coordinated, safety-oriented, interdisciplinary teamwork. As such, strengthening safety culture and team processes represents a core component of system-level strategies examined in this systematic review on safety culture and multidisciplinary teamwork in paediatric care transitions.

Taitz [7] similarly emphasises the importance of developing a culture of safety and reliability in paediatric healthcare across multiple organisational levels, from individual wards and clinical units to hospitals and wider healthcare systems. The author notes that quality and safety programmes may remain fragmented across healthcare systems, with variable outcomes, highlighting the importance of organisational leadership and system-wide approaches to patient safety.

Leadership commitment, structured teamwork practices, shared situational awareness, and standardised procedures are identified as essential components for creating an environment in which multidisciplinary teams can reliably perform complex tasks such as medication reconciliation. By underscoring that cultural and organisational alignment is as critical as clinical protocols themselves, these findings reinforce the need for integrated, system-wide strategies to support safe paediatric care transitions.

Despite international recommendations promoting standardized medication reconciliation practices, wide variability in implementation remains common across healthcare systems. Barriers such as inconsistent workflows, variable staff engagement, insufficient training, and limited integration of pharmacy expertise can reduce the quality and reliability of the process. Consequently, there is an ongoing need to examine which approaches most effectively enhance the accuracy of medication lists and reduce preventable medication errors in paediatric transitions of care.

The aim of this systematic review is to synthesise existing evidence on the associations between safety culture, leadership support, multidisciplinary teamwork, medication accuracy, and the safety of paediatric care transitions across diverse healthcare settings.

## 2. Methods

This systematic review was conducted in accordance with the Preferred Reporting Items for Systematic Reviews and Meta-Analyses (PRISMA 2020) guidelines [8]. This review involved the construction of the research question, specification of the criteria for study inclusion and exclusion, systematic literature searching, selection of studies, evaluation of the methodological quality of selected studies, data extraction, and narrative synthesis.

The research question was developed using the PIO (Population, Intervention/Exposure, Outcome) framework:

What evidence exists regarding the role of organisational safety culture and team-based approaches in medication safety and accuracy during paediatric care transitions?

The research question follows the PIO framework presented in Table 1, and the eligibility criteria for study selection are presented in Table 2.

### 2.1. Literature Search Strategy

A systematic literature search was conducted in PubMed from database inception to 31 August 2026. The search strategy was developed according to the review question and predefined eligibility criteria and combined terms related to the paediatric population, transitions of care, medication safety and medication reconciliation, and organisational or team-based approaches. Keywords were combined using the Boolean operators AND and OR. Search terms included combinations of child, paediatric/pediatric, adolescent, infant, medication reconciliation, medicines reconciliation, medication discrepancy, medication error, medication safety, transition of care, admission, transfer, handoff/handover, discharge, pharmacist, multidisciplinary, interdisciplinary, interprofessional, teamwork, communication, safety culture, and organisational/organizational. The search was restricted to studies published in English. The complete PubMed search strategy is provided in the Supplementary Materials.

The search terms were combined using the Boolean operators OR and AND. Synonymous terms within each concept were combined using OR, whereas the four main concepts (population, care transition, medication safety, and organisational/team-based approaches) were combined using AND. The complete PubMed search strategy was as follows:

(child[Title/Abstract] OR pediatric[Title/Abstract] OR paediatric*[Title/Abstract] OR adolescent*[Title/Abstract] OR infant*[Title/Abstract]) AND (“transition of care”[Title/Abstract] OR “transitions of care”[Title/Abstract] OR admission[Title/Abstract] OR discharge[Title/Abstract] OR transfer*[Title/Abstract] OR handoff*[Title/Abstract] OR handover*[Title/Abstract]) AND (“medication reconciliation”[Title/Abstract] OR “medicines reconciliation”[Title/Abstract] OR “medication discrepancy”[Title/Abstract] OR “medication discrepancies”[Title/Abstract] OR “medication error”[Title/Abstract] OR “medication errors”[Title/Abstract] OR “medication safety”[Title/Abstract]) AND (pharmacist*[Title/Abstract] OR multidisciplinary[Title/Abstract] OR interdisciplinary[Title/Abstract] OR interprofessional[Title/Abstract] OR teamwork[Title/Abstract] OR communication[Title/Abstract] OR “safety culture”[Title/Abstract] OR organisational[Title/Abstract] OR organizational[Title/Abstract])**.

### 2.2. Study Selection

Study selection was conducted in two stages. Titles and abstracts were initially screened against the predefined eligibility criteria, followed by full-text assessment of potentially relevant studies. The screening and study selection were conducted by one reviewer, with eligibility decisions discussed with the second author where appropriate. Studies meeting the predefined inclusion criteria were included in the final review.

### 2.3. Quality Appraisal

The methodological quality of the included studies was assessed using the Mixed Methods Appraisal Tool (MMAT), version 2018 [9]. Each study was appraised using the MMAT criteria corresponding to its study design. In accordance with MMAT guidance, an overall methodological quality score was not calculated.

### 2.4. Narrative Synthesis

Considering the heterogeneity in the study designs, settings, measurement tools, and outcomes, a narrative synthesis approach was used to integrate the findings of the included studies. Key themes were developed through several iterations based on the study’s aim, the methods description, and the effects on medication accuracy and the transition’s safety.

## 3. Results

**Figure 1:**
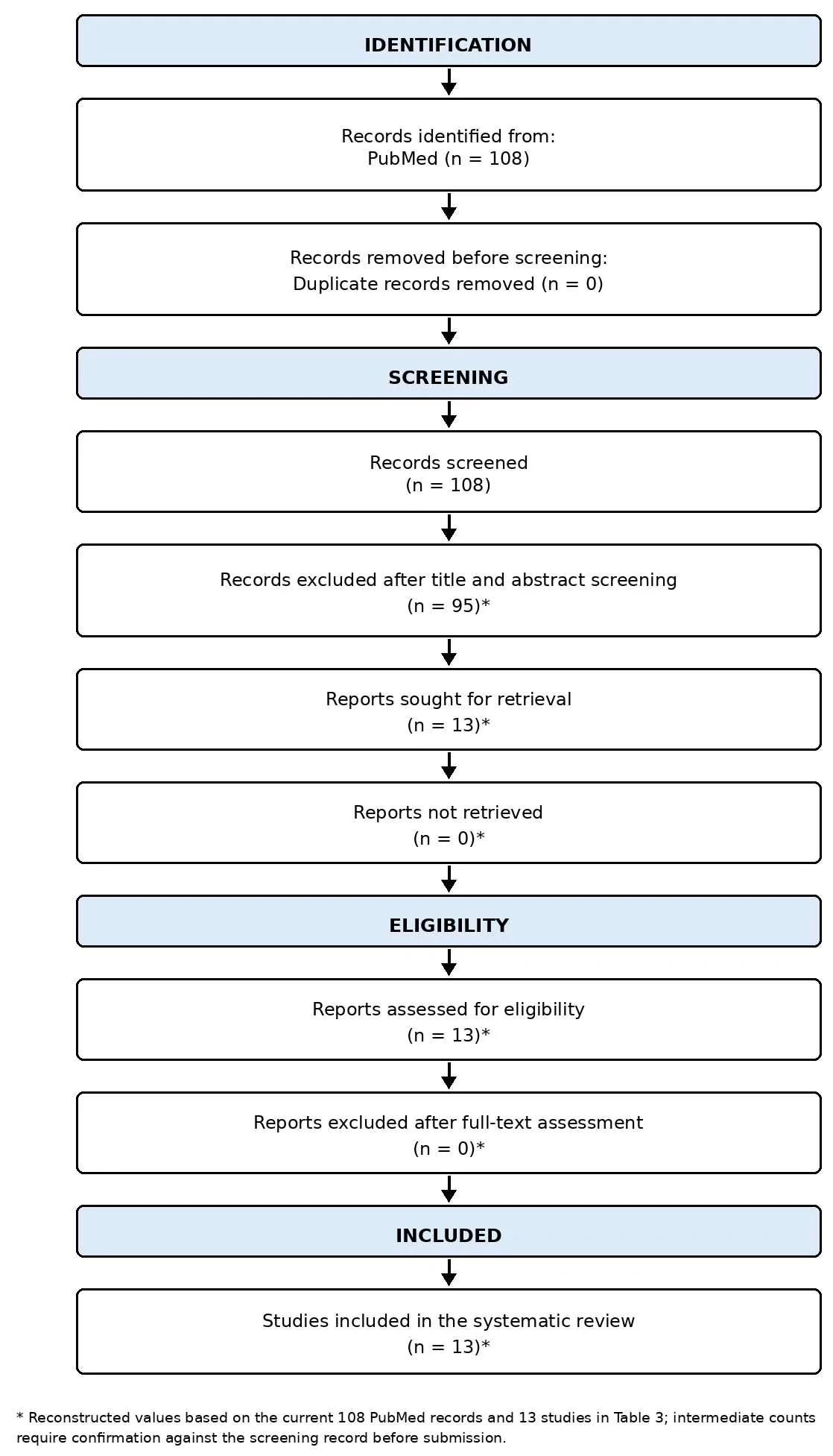
PRISMA 2020 Flow Diagram of Study Selection Process.

A total of 108 results were obtained from the search conducted on PubMed. After reviewing titles and abstracts, 95 reports were excluded. The remaining 13 reports were selected for full-text evaluation. All of these 13 reports satisfied our inclusion criteria and were selected for the narrative synthesis. The process of study selection is reported in the PRISMA 2020 flow diagram (Figure 1).

### 3.1. Study Characteristics

The 13 included studies used heterogeneous methodological approaches, including prospective observational studies, retrospective chart reviews, pilot studies, and quality-improvement initiatives. The studies were conducted predominantly in paediatric hospital settings and examined medication safety at key transitions of care, including hospital admission, intrahospital transfer, discharge, and hospital-to-home transitions. Across the included studies, the most frequently evaluated approaches involved pharmacist-led or pharmacist-supported medication reconciliation, multidisciplinary medication-safety interventions, and structured processes for improving the accuracy and completeness of medication information. The characteristics of the included studies are summarised in Table 3.

The characteristics of the included studies are summarized in Table 3.

### 3.2. Methodological Quality of the Included Studies

We assessed the methodological quality of the 13 included studies using the Mixed Methods Appraisal Tool (MMAT), version 2018 [9]. We evaluated each study using the MMAT criteria corresponding to its methodological design. We considered the two initial screening questions before applying the five design-specific criteria. Individual criteria were rated as “Yes”, “No”, or “Can’t tell” when the information reported in the study was insufficient to permit a definitive judgment. In accordance with MMAT guidance, we did not calculate an overall methodological quality score. Table 4 presents the criterion-level methodological appraisal of the included studies.

Overall, the methodological appraisal showed variation across the included studies in how fully they met individual MMAT criteria. We considered methodological strengths and limitations when interpreting the findings and incorporated them into the narrative synthesis.

### 3.3. Integration of Included Studies

Despite variation in study design, transition setting, interventions, and outcomes related to medication safety, the included studies’ findings were described synthetically. Most evidence related to medication reconciliation, pharmacist involvement, multidisciplinary teams, structured communication, and standardized medication-safety processes and procedures in pediatric care transitions. While the themes that emerge are not intended to be explanatory, the findings are presented as recurrent themes in the studies in question.

Medication reconciliation and pharmacist intervention were the most studied approaches in the included studies. Pharmacist-driven or pharmacist-supported interventions improved identification of medication discrepancies, completion of medication histories, and accuracy of admission and discharge medications. Several studies evaluated improvements in transferring medication information to other healthcare professionals in their care settings. They implemented standardized processes or structures.

The medication safety interventions focused on involving multiple disciplines and using specific communication structures. The results suggested that clear workflows, defined professional roles, and consistent medication-information exchange during pediatric care transitions could improve the safety of care transitions. The individual approaches to these interventions, settings, and measures of outcomes were very different, and limited direct comparisons among them.

Improved medication safety during pediatric care transitions was noted with the adoption of structured medication safety processes involving multiple disciplines and professional roles. The evidence should not be generalized because the studies were not randomized.

## 4. Discussion

This systematic review finds that medication safety is a critical concern when dealing with the transition of pediatric care, particularly at admission, transfer, and discharge. The studies included reported discrepancies and incomplete or incorrect medication information at various transition stages. The most common strategies used to mitigate the identified risks included the participation of pharmacists, structured medication reconciliation, collaboration of multiple professions, and the use of standardized communication.

While many studies noted positive effects of the measures on either the process or the outcome of medication, the evidence was of poor quality because of differences in study design, clinical setting, intervention characteristics, and outcome measurement. Most evidence was non-randomized, limiting the ability to draw conclusions and directly compare different measures. Therefore, the results should be viewed as showing that certain measures may create positive changes, rather than comparing measures to one another and identifying the best approach.

In the heterogeneous evidence base, there appeared to be both organizational and team factors in the implementation of medication-safety processes in the context of paediatric care transitions. However, direct evidence on organizational safety culture was limited. The studies included in the review focused more on pharmacist participation, medication reconciliation, and structured communication. Therefore, the findings reinforce the need for an organization- and team-based approach to medication safety. However, they do not provide an independent or causal effect of safety culture on medication-related outcomes.

One of the most frequently studied team-based methods in the included studies was the inclusion of pharmacists. Medication reconciliation led or supported by a pharmacist improved the identification of discrepant medications, more complete medication records, and more accurate medication records during the paediatric care transition. These results suggest that pharmacists may contribute to patient safety by spotting clinically relevant discrepancies and promoting reconciliation of admissions, transfers, and discharges. Because of differences in the design of the studies, interventions, and endpoints, no substantive comparisons can be drawn to the relative effectiveness of the different pharmacist-led approaches.

Structured interprofessional communication and clear workflow definitions may further support medication safety by promoting the consistent exchange of medication information among health care professionals [22].

In addition to interpersonal communication, the structural aspect of information management plays a role in information consistency. The clinical documentation and medical records may be limited by a lack of structure and flexibility [23]. Vanalli et al. [24] indicated that unstructured nursing activities can be identified and standardized using natural language processing and semantic mapping, suggesting that consistent documentation of clinical information is possible if nursing activities are done using standardized terminologies.

From a systems perspective, the SEIPS model by Carayon et al. [25] describes the interactions of health care professionals, tasks, technologies, and the physical and social environments of the organization and how they affect the processes of care and patient safety. This model describes a number of limitations that are helpful in interpreting the study results. However, it should be noted that this model is a description and an extension of existing literature on patient safety, and should not be considered part of the empirical evidence from the included studies.

Medication safety during pediatric care transitions is supported by structured medication reconciliation, the inclusion of pharmacists, collaboration across disciplines, and consistent communication of medication information. Organizational factors may influence the use of these strategies, but the literature currently available does not allow separation of the strategies from the factors that influence their use. More advanced research is required to evaluate which organizational and team strategies provide the greatest level of safety when used across multiple pediatric transition settings. These studies should utilize more sophisticated and well defined outcome measures related to medication safety.

### 4.1. Implications for Practice and Policy

This review advances knowledge of clinical practice and policy related to medication safety for children across care transitions.

First, pharmacist participation would be beneficial at multiple medication-reconciliation points, particularly at high-risk times such as hospital admission or discharge.

Second, medication reconciliation at high-stakes times during pediatric care transitions would benefit from standardized, consistent processes.

Third, incorporating interprofessional communication and standardized professional roles during high-stakes care transitions would foster communication among healthcare professionals about patients’ medication needs.

Fourth, healthcare organizations are responsible for assessing local, clinical, and operational needs to implement effective medication safety interventions, even if they differ from those of other organizations.

Fifth, at the policy and organizational level, efforts to increase pediatric medication safety should foster and sustain interprofessional collaboration and the inclusion of pharmacists, as well as standardized transition-of-care processes. However, more sophisticated research is needed to determine which organizational models are better.

At this time, with the limited and mostly non-randomized evidence, the safe incorporation of structured medication safety processes at paediatric care transitions should be considered as best approaches to inform practice, but should not be viewed as definitive.

### 4.2. Limitations

This review has some limitations. First, the review protocol was not available for prospective registration. To improve methodological clarity and transparency and reduce the likelihood of bias, we aligned each review step with the PRISMA 2020 statement and defined it in line with preset review criteria.

The included studies showed high heterogeneity due to differences in design, clinical setting, point of transition, intervention characteristics, and medication safety outcome measures. In most cases, the studies were non-randomized. Because of this heterogeneity, we could not conduct a quantitative synthesis, so we conducted a narrative synthesis.

Studies used different outcome measures. Measures included medication discrepancies, accuracy of medication history, errors during reconciliation, and other safety-related measures. Heterogeneity in outcome measures meant the synthesis could not be quantified, only described narratively.

Because of differences in clinical settings and study organization, the findings may not generalize to other settings.

Language bias was possible because we included only studies published in English.

We also had only one reviewer for study selection, meaning there was no second reviewer to check for bias in selection. This is also something to consider when interpreting the results of this review.

Furthermore, we searched only PubMed; therefore, we may have missed other relevant studies not indexed in PubMed. This is also something to consider when interpreting the search results.

## 5. Conclusions

This systematic review shows the potential for pharmacists to improve paediatric medication safety during care transitions through their involvement in different aspects of the medication-safety process. These include structured medication reconciliation, active participation of multiple healthcare professionals, and planned communication. The included studies reported better medication accuracy, improved identification of medication discrepancies, and more complete medication information during admission, transfer, and discharge when these approaches were implemented.

The strength of the available evidence was limited, mainly because of differences and limitations in study design. Many of the included studies did not use random allocation; therefore, firm conclusions about the effectiveness of individual strategies cannot be made, nor can these strategies be ranked according to their effectiveness. Evidence specifically addressing the role of organisational safety culture was also limited. Further comparative studies are needed to determine which approaches are most effective and how they can be implemented and sustained across different paediatric healthcare settings.

## Figures and Tables

**Table 1:** Population–Intervention–Outcome (PIO) framework used to formulate the research question.

	Description
Population	Paediatric patients aged 0–18 years undergoing transitions of care, including admission, transfer, handover, or discharge.
Intervention/Exposure	Organisational and team-based approaches, including safety culture initiatives, multidisciplinary teamwork, interprofessional communication, pharmacist involvement, and structured medication reconciliation during paediatric care transitions.
Outcome	Medication accuracy, medication discrepancies, reconciliation errors, medication errors, adverse drug events, and other medication-safety outcomes during paediatric care transitions.

**Table 2:** Inclusion and exclusion criteria.

	Inclusion Criteria	Exclusion Criteria
Population	Children and adolescents (0 to 18 years old) receiving healthcare during transitions of care	Adult populations or studies in which paediatric data cannot be identified separately
Transition of care	Admission, transfer between units or healthcare settings, clinical handover, or discharge	Studies not involving an identifiable paediatric transition of care
Intervention/Exposure	Organisational or team-based approaches related to medication safety, including medication reconciliation, pharmacist involvement, multidisciplinary or interprofessional collaboration, structured communication, and safety-related organisational processes.	Approaches unrelated to medication safety or paediatric transitions of care
Outcome	Medication accuracy, medication discrepancies, reconciliation errors, medication errors, adverse drug events, or other medication-safety outcomes during paediatric care transitions	Studies without a medication-safety or medication-reconciliation outcome related to a paediatric care transition
Study type	Randomized controlled trials, quasi-experimental studies, prospective or retrospective observational studies, cross-sectional studies, qualitative studies, and mixed-methods studies	Literature reviews, systematic reviews, meta-analyses, editorials, commentaries, conference abstracts, protocols, opinion papers, and duplicate publications
Language	English	Languages other than English

**Table 3:** Characteristics of the studies included in the systematic review.

Author(s), Year	Country/Setting	Design	Population/Context	Transition of Care	Intervention/Exposure	Medication-Safety Outcome	Key Findings
Gunkelman et al. [4], 2024	USA; freestanding children’s teaching hospital	Quality-improvement study	Children admitted to the paediatric hospital medicine service	Hospital admission	Multidisciplinary medication-reconciliation improvement using EHR workflow simplification, resident education, chart audits, real-time feedback, and accountability	Accuracy of the home medication list, assessed using medication removal as a proxy measure	The proportion of patients with ≥1 medication removed from the home medication list increased from 35% to 48%, and the improvement was sustained for 12 months.
Hovey et al. [10], 2023	USA; children’s hospital	Observational	283 admission medication reconciliations	Hospital admission	Pharmacist-led medication reconciliation	Pharmacist interventions and prevention of adverse drug events	69% of reconciliations required intervention; interventions affected inpatient care in 21.9% of patients, and eight major adverse drug events were prevented.
Stone et al. [11], 2010	USA; tertiary children’s hospital	Prospective quality-improvement cohort study	23 medically complex children identified from 219 admissions	Hospital admission	Medication reconciliation using five medication-information sources	Medication discrepancies and admitting-order errors	39 errors were identified among 182 admission medications (21%), affecting 13 patients (57%); five errors were considered potentially serious or life-threatening.
Provine et al. [12], 2014	USA; paediatric institution	Prospective single-centre pilot study	100 medication histories	Hospital admission	Pharmacist-managed medication history and reconciliation with the medical team	Medication discrepancies	Pharmacists identified and corrected 309 discrepancies; 30 clinically pertinent discrepancies required intervention and communication with the medical team.
Huynh et al. [13], 2016	UK; paediatric hospital	Prospective review	142 paediatric discharge episodes	Hospital discharge to community	Pharmacist review and medicines reconciliation of discharge medication information	Medication discrepancies at discharge	Medication discrepancies were frequently identified in discharge documentation, demonstrating the importance of pharmacist review at the hospital-to-community transition.
Gattari et al. [14], 2015	USA; tertiary children’s hospital	Prospective observational study	69 paediatric patients	Hospital discharge	Medication reconciliation and comparison of discharge medication documentation	Medication discrepancies at discharge	Medication discrepancies were identified in 26% of patients at discharge, demonstrating the risk of medication-information inconsistencies during the transition from hospital care.
Alcântara et al. [15], 2021	Brazil; paediatric hospital	Prospective observational study	114 paediatric patients	Admission, internal transfer, and discharge	Medication reconciliation across transitions of care	Unintentional medication discrepancies	Unintentional medication discrepancies were identified across paediatric transitions of care; 74.5% of patients had at least one discrepancy, highlighting the vulnerability of medication information during transitions.
Aires-Moreno et al. [16], 2021	Brazil; four teaching hospitals	Prospective observational multicentre study	248 children aged 1 month–12 years	Admission, transfer, and discharge	Medication reconciliation and assessment of medication discrepancies across transition points	Intentional and unintentional medication discrepancies and potential clinical harm	20.2% of patients had at least one unintentional discrepancy; medication omission was the most frequent unintentional discrepancy (54.1%), and some discrepancies had the potential to cause moderate or severe harm.
Alcântara et al. [17], 2022	Brazil; paediatric department of a public hospital	Quality-improvement implementation	Paediatric patients aged 28 days–12 years; three PDSA cycles (*n* = 34, *n* = 35, *n* = 30)	Admission, intrahospital transfer, and discharge	Implementation of medication reconciliation using the Model for Improvement and PDSA cycles with healthcare-team involvement	Medication discrepancies and discrepancy resolution	Across the three cycles, 72, 32, and 24 discrepancies were identified, with resolution rates of 90.3%, 96.8%, and 100%, respectively.
Hernandez et al. [18], 2024	USA; large academic children’s hospital	Single-centre quality-improvement initiative	60 high-risk paediatric patients	Hospital discharge	Pharmacist-driven discharge medication reconciliation and counselling supported by pharmacy, medical, and IT teams	Pharmacist interventions related to medication reconciliation and discharge medication safety	Pharmacists completed medication reconciliation and/or counselling for 60 patients and performed 65 interventions. All pharmacist recommendations were accepted and implemented by prescribing providers.
Lingle et al. [19], 2023	USA; children’s hospital/complex care clinic	Retrospective chart review	92 encounters involving 60 children with medical complexity	Hospital-to-home discharge transition	Pharmacist involvement in discharge medication evaluation and medication reconciliation	Pharmacist medication-reconciliation and clinical interventions	Pharmacists made 283 interventions across 92 encounters; 91 (32%) were medication-reconciliation interventions, and 82 encounters (89%) required at least one pharmacist intervention.
Ring et al. [20], 2023	USA; Children’s National Hospital	Quality-improvement study	Paediatric cardiovascular surgery and blood and marrow transplant patients	Hospital discharge and first outpatient follow-up	Interdisciplinary implementation of an EHR-integrated medication discharge platform (MedActionPlanPro)	Discharge medication reconciliation accuracy and medication matching	Integration of the medication platform increased medication matching by 25.6% from baseline to post-intervention (*p* < 0.001) and was associated with improved discharge medication reconciliation safety and provider efficiency.
Sheth et al. [21], 2024	USA; paediatric ED and four urgent care centres	Quality-improvement study	Paediatric patients discharged from the ED or urgent care centres	Discharge	Multidisciplinary standardisation of medication reconciliation using EHR optimisation, clinical decision support, education, training, and provider feedback	Completion of medication reconciliation and home-medication review	Medication reconciliation increased from 35% to 82% in urgent care and from 26% to 64% in the ED; home-medication review also improved substantially following implementation.

**Table 4:** Methodological quality assessment of the included studies using the Mixed Methods Appraisal Tool (MMAT, 2018).

Study	MMAT Design Category	S1	S2	C1	C2	C3	C4	C5
Stone et al. [11], 2010	Quantitative non-randomized	Y	Y	CT	CT	CT	CT	CT
Provine et al. [12], 2014	Quantitative non-randomized	Y	Y	CT	CT	CT	CT	CT
Huynh et al. [13], 2016	Quantitative descriptive	Y	Y	CT	CT	CT	CT	CT
Hovey et al. [10], 2023	Quantitative non-randomized	Y	Y	CT	CT	CT	CT	CT
Gattari et al. [14], 2015	Quantitative descriptive	Y	Y	CT	CT	CT	CT	CT
Alcântara et al. [15], 2021	Quantitative descriptive	Y	Y	CT	CT	CT	CT	CT
Aires-Moreno et al. [16], 2021	Quantitative descriptive	Y	Y	CT	CT	CT	CT	CT
Alcântara et al. [17], 2022	Quantitative non-randomized	Y	Y	CT	CT	CT	CT	CT
Hernandez et al. [18], 2024	Quantitative non-randomized	Y	Y	CT	CT	CT	CT	CT
Lingle et al. [19], 2023	Quantitative descriptive	Y	Y	CT	CT	CT	CT	CT
Ring et al. [20], 2023	Quantitative non-randomized	Y	Y	CT	CT	CT	CT	CT
Gunkelman et al. [4], 2024	Quantitative non-randomized	Y	Y	CT	CT	CT	CT	CT
Sheth et al. [21], 2024	Quantitative non-randomized	Y	Y	CT	CT	CT	CT	CT

Abbreviations: MMAT, Mixed Methods Appraisal Tool; S1 = Are there clear research questions? S2 = Do the collected data allow the research questions to be addressed? For quantitative non-randomized studies: C1 = participants representative of the target population; C2 = appropriate measurements of outcome and intervention/exposure; C3 = complete outcome data; C4 = confounders accounted for in the design and analysis; C5 = intervention administered/exposure occurred as intended. For quantitative descriptive studies: C1 = sampling strategy relevant to the research question; C2 = sample representative of the target population; C3 = appropriate measurements; C4 = low risk of nonresponse bias; C5 = statistical analysis appropriate to answer the research question. Y = Yes; CT = Can’t tell. No overall methodological quality score was calculated.

## Data Availability

No new data were created or analyzed in this study. Data sharing is not applicable to this article.

## Supplementary Materials

The following supporting information can be downloaded at: https://www.mdpi.com/article/10.3390/children13101387/s1.
